# Knowledge, Attitudes, and Practices of Healthcare Workers in Jordan towards the COVID-19 Vaccination

**DOI:** 10.3390/vaccines10020263

**Published:** 2022-02-09

**Authors:** Lujain Lataifeh, Abdallah Al-Ani, Isam Lataifeh, Khawlah Ammar, Ameera AlOmary, Fawzi Al-hammouri, Maysa Al-Hussaini

**Affiliations:** 1 School of Medicine, Jordan University of Science and Technology, Irbid 21110, Jordan; Llataifeh@yahoo.com (L.L.); ILataifeh@khcc.jo (I.L.); 2 Office of Scientific Affairs and Research, King Hussein Cancer Center, Amman 11942, Jordan; abdallahalany@gmail.com (A.A.-A.); KA.11148@khcc.jo (K.A.); 3 Department of Surgery, King Hussein Cancer Center, Amman 11942, Jordan; 4 Department of Obstetrics and Gynecology, Islamic Hospital, Amman 11942, Jordan; rawaahf@yahoo.com; 5 Department of Pediatrics, The Specialty Hospital, Amman 11942, Jordan; fhammouri@gmail.com; 6 Department of Pathology and Laboratory Medicine, King Hussein Cancer Center, Amman 11942, Jordan; 7 Human Research Protection Program Office, King Hussein Cancer Center, Amman 11942, Jordan

**Keywords:** hesitancy, healthcare workers, females, Jordan

## Abstract

The rapid development of COVID-19 vaccines raises concerns over vaccine hesitancy among healthcare workers (HCWs) and the general public, which made understanding the factors influencing hesitancy crucial in the maintenance of a solid healthcare system. This cross-sectional study investigated the knowledge, attitudes, and perceptions (KAP) of Jordanian HCWs to the COVID-19 vaccine from February to March 2021, using a self-administered questionnaire validated by a panel of public health experts. A total of 364 Jordanian HCWs were included in the final analysis, in which women accounted for 48.8% of the total sample. HCWs subjected to the seasonal flu vaccine were significantly more likely to uptake the COVID-19 vaccine. In comparison to nurses, physicians were significantly more likely to take or register for the vaccine. They demonstrated significantly higher knowledge of the vaccine’s effectiveness, side effect profile, recommended doses, and target population. Among our participants, the most common reasons for vaccine hesitancy include a lack of confidence, inadequate knowledge, and disbelief in effectiveness. Vaccine hesitancy among Jordanian HCWs is low, with discrepancies between nurses and physicians. It is pertinent for independent committees and trusted authorities to provide interventions and raise awareness regarding the vaccine’s safety and efficacy.

## 1. Introduction

Vaccination has resulted in the control of many infections, some of which used to be deadly or caused permanent disability [1]. The publishing of the Wakefield study, linking the mumps, measles, and rubella (MMR) vaccine with the risk of autism, has halted many parents from vaccinating their children for years, thus potentially jeopardizing their health as well as others’ [2,3]. In other instances, parental delay of child vaccines was related to conspiratorial thinking and needle sensitivity [4]. The seasonal flu vaccines have also been reported as underutilized, with ethical debates on the morality of refusing to receive the vaccine [5]. How people perceive vaccine safety and the social influences might be important determinants of accepting any vaccine [6]. Applying psychologically-, rather than intuitively-, based persuasive methods might prove to be a more effective strategy to overcome vaccine hesitancy [7]. 

Amid the current COVID-19 pandemic and in view of the recently available vaccines, the same debate is heated again, probably on a larger scale [8]. In the context of a pandemic, vaccine hesitancy is a major barrier to implementing vaccination campaigns [9]. Amongst healthcare workers (HCWs), vaccine hesitancy is primarily influenced by providers’ confidence, risk perception, and concerns regarding the safety and efficacy of vaccines [10,11,12,13]. These factors are context-specific, complex, and multidimensional; they are often affected by broader organizational, political, cultural, and historical factors [10]. Thus, developing tailored strategies to address concerns to decrease vaccine hesitancy will be the key to success. 

In terms of the COVID-19 vaccine, studies demonstrated favorable attitudes from HCWs; however, many also showed significant HCWs concerns primarily due to inadequate knowledge, a lack of trust, and the presence of anti-vaccine media [14,15]. From a layman perspective, in addition to lack of knowledge towards the vaccine, religiosity may hinder vaccine acceptance [16,17]. The association of vaccines with abortion-derived fetal cell lines or pork may induce moral concerns in certain communities characterized by Islam or Christian Catholicism, thus prioritizing religious healing over medicine among devotees [16].

In Jordan, the perception of the public in general and healthcare workers in particular regarding the benefits of the vaccination, especially the COVID-19 vaccine, is of utmost importance as the uptake of the vaccine is still below the anticipated and accepted levels, especially that the country now has entered into this third wave of the pandemic [18]. In a previous study in 2016, which investigated the predictors of uptake of seasonal influenza vaccine among HCWs in Jordan, only 51.6% of participants ever had the influenza vaccine vs. 32.1% who received the influenza vaccine in the past year. The study also showed that past vaccination behavior and the perceived benefit scale were the only significant predictors of intentions to vaccinate against influenza in the next season [19]. A recent study of the public perception of the COVID-19 vaccine in Jordan showed that only 29.6% had or will take the seasonal influenza vaccine, and only 28.4% of participants will take the COVID-19 vaccine when available [20]. The Jordanian government started its vaccine rollout program in January 2021, primarily targeting HCWs and the elderly. The program was continuously expanded to include adolescents as old as 16 years. Jordan has also utilized its National Defense Law number 32 to mandate vaccination as means to protect the public’s health. At the current moment, vaccination trackers (i.e., an application that proves vaccination status) are required in all governmental and most private institutions.

In this study, the aim is to investigate Jordanian HCWs knowledge, attitudes, and practices (KAP) toward the COVID-19 vaccine. Moreover, we explored if perceptions towards the seasonal flu vaccine affected HCWs attitudes towards the COIVD-19 vaccine. Ultimately, we sought to compare our findings to that of the international literature to provide a valid frame of reference.

## 2. Materials and Methods

An anonymous, self-administered English-language structured questionnaire was distributed during February and March 2021 in two private hospitals in Amman, the capital city of Jordan. The questionnaire consisted of three domains, including demographics, previous experience with seasonal flu vaccines, and the COVID-19 vaccine. Demographics included age, gender, marital status, occupation within the HCW sector, and years of experience. The second domain consists of three questions on previous experience and two questions on previous practices related to the seasonal flu vaccine. An additional question on the perception of administering the seasonal flu vaccine to all HCWs was also included. The third domain consisted of 20 questions that examined KAP with regards to the COVID-19 vaccine. A 4-tier Likert scale (strongly agree, agree, disagree, strongly disagree) was used for most questions. For the purpose of analysis, the answers were grouped into two categories (agree including the strongly agree and agree, versus disagree including disagree and strongly disagree). The questionnaire’s content validity was ensured by a panel of public health and epidemiology experts and was subsequently piloted on 10 HCWs to ensure clarity and attain relevant suggestions. Two modalities for the distribution of the questionnaire were used; an online survey using SurveyMonkey and hard copy distribution. The answers were then entered into the same SurveyMonkey form using the data entry option as to maintain the data set free of errors.

A cross-sectional convenient sample was determined to suit the purpose of the study. It was estimated that 341 HCWs would be needed to give the needed power for the study (95.0% confidence) [21]. The questionnaire was distributed among HCWs in almost all departments and divisions of the selected hospitals. HCWs included physicians and nurses, including midwives.

Data were then exported to an SPSS (version 26) file, coded, and analyzed. Descriptive analysis was used to report HCW responses. Frequencies and percentages were used for the categorical variables, and associations were measured between categorical variables using the Chi-square test. The *p*-value and adjusted *p*-value for occupation and gender were calculated for most questions using binary logistic regression. A *p*-value of ≤0.05 was considered significant. 

The study was approved by the Institutional Review Boards of the participating institutions. We requested a waiver of documentation of the informed consent. A cover page explaining the purpose and the voluntary nature of the study was used instead.

## 3. Results

Out of 400 distributed questionnaires, 364 surveys were included in the final analysis, including 215 (59.1%) from physicians and 149 (40.9%) from nurses and midwives. The mean age across the sample was 36 ± 12.8 years, in which 57% of participants were younger than 30 years of age. There was no significant difference in the age groups distribution between physicians and nurses. There was a balanced representation of males and females among physicians (51.2% vs. 48.8%, respectively), whereas, in nursing, males were outnumbered by females (16.8% vs. 83.2%, respectively; *p*-value < 0.001). The distribution of other demographic variables, including marital status and years of experience, were not significantly different between both groups. Table 1 summarize the demographics of the studied cohort.

There was no statistical difference in relation to practices between physicians and nurses regarding their previous administration of the seasonal flu vaccine (*p*-value = 0.460), nor in reporting adverse events, as only a minority reported any (*p*-value = 0.099) (Refer to Table 1).

There was a significant association between HCWs who administered the seasonal flu vaccine and the intention to uptake the COVID-19 vaccine once available (*p*-value = 0.032). Similarly, this association was also present with the registration on the special platform for the COVID-19 vaccine (*p*-value = 0.002). When adjusting for gender, physicians were more inclined to take the vaccine once it becomes available (adjusted *p*-value < 0.001) and to have already registered in the special COVID-19 platform (adjusted *p*-value < 0.001). Gender and occupation were not significantly associated with a difference in regular seasonal vaccination (*p*-value = 0.158 and 0.489, respectively). The observation also exists for experiencing adverse effects related to the season (*p*-value = 0.099 and 0.471, respectively).

For the 190 participants that have registered for the special COVID-19 vaccine platform, the most common reasons were to protect oneself and family members from the virus (61%), fear of becoming infected (18.4%), and enforced institutional rules and regulations (15.2%). Conversely, the most common reasons for those not registering on the platform (*n* = 155) were a lack of confidence in the vaccine (26.5%), a lack of adequate knowledge to decide whether to administer the vaccine (20.0%), and the disbelief in its effectiveness (18.7%). 

More physicians, in comparison to nurses, believed they would register for the COVID-19 vaccine (*p*-value ≤ 0.001), and they would take the COVID-19 vaccine once it becomes available (*p*-value < 0.001). In addition, more physicians, in comparison to nurses, believed that the seasonal flu (*p*-value = 0.002) and COVID-19 vaccine (*p*-value = 0.001) should be given to all HCWs. Physicians were more inclined to recommend the COVID-19 vaccine to eligible individuals compared to nurses (*p*-value < 0.001). All of which remained statistically significant after adjustment for gender (refer to Table 2). In addition, more physicians believed they had sufficient information about the vaccine compared to nurses (*p*-value = 0.001). For those who believed they did not have sufficient information regarding the vaccine, there was no difference between physicians and nurses towards the need to attend sessions to increase their knowledge (*p*-value = 0.382).

When asked to specify the area in which more information is needed, there was no difference between both groups in relation to the need for more information related to the safety (*p*-value = 0.933), efficacy (*p*-value = 0.326), and duration of protection of the vaccine (*p*-value = 0.399), although there was a difference in relation to the need of more information related to the cost demonstrated by nurses (*p*-value = 0.022).

In terms of gender, females were less likely to have sufficient information about the vaccine (*p*-value < 0.001). However, there was no statistical difference in being concerned that themselves or a family member could become infected with COVID-19 (adjusted *p*-value = 0.203), and the need to attend sessions related to increasing awareness regarding COVID-19 (adjusted *p*-value = 0.615) (Refer to Table 3).

In terms of knowledge, our results demonstrate that physicians are more knowledgeable, compared to nurses, about the effectiveness of the vaccine in preventing COVID-19 infection and its related serious morbidity and mortality (*p*-value < 0.001), that two doses should be given to prevent infection (*p*-value < 0.001), and that the vaccine would not cause serious side effects (*p*-value < 0.001). Physicians had more knowledge in relation to the need to vaccinate previously infected individuals (*p*-value = 0.003). Physicians and nurses were divided almost equally on whether the vaccine will lead to a long-lasting immunity (*p*-value = 0.516). Both groups also believed that vaccination would not alleviate the need for protective measures (*p*-value = 0.103). These associations remained consistent after adjusting for gender (refer to Table 2 and Table 3).

## 4. Discussion

Our results demonstrate that among HCWs, physicians display more positive attitudes towards seasonal flu and COVID-19 vaccinations. Similarly, in comparison to their nursing counterparts, physicians have more knowledge about the COVID-19 vaccine in terms of effectiveness, dose efficacy, and adverse effect profile. On the other hand, females perceived a lower fund of knowledge about COVID-19 and portrayed lower levels of transmission concerns and a propensity to avoid vaccinating already infected individuals.

Across the literature, COVID-19 vaccine acceptance rates among HCWs range from 20.0% to 94.0% [15,22,23,24,25,26,27,28,29,30,31,32,33,34,35,36,37,38], in which European nations (Germany, France, Poland, and Italy) [26,28,34,38], Canada [23], Turkey [37], and China [36,39] had high rates starting from about 70%, while Middle Eastern and African countries consistently demonstrated lower rates below 50% [22,24,25,29,31,33]. The higher propensity of European HCWs to accept COVID-19 vaccination in comparison to their Middle Eastern and Asian counterparts was demonstrated in a recent meta-analysis [40]. Nonetheless, an Iraqi/Kurdish cohort of 1704 HCWs showed vaccine hesitancy as low as 27.9% [41]. Among our participants, COVID-19 vaccine acceptance ranges from 83.3% for physicians to 42.6% for nurses. While holding a significant variability between physicians and nurses, these rates are higher than what was reported in a large multicentric study on Arab HCWs (26.7%), in which Jordanian HCWs reported a 21.8% acceptance rate [33]. Such discrepancy among Jordanian reports might be attributed to sampling bias, barriers towards vaccination, the different time frames of cross-sectional reports, or, more recently, the enhancement of vaccination [42], especially among HCWs. Reports for a variety of countries, including Saudi Arabia (25–65%) [32,43], Egypt (21–26%) [24,25], and the USA (36–57.5%) [15,44], showed similar degrees of variance, which were mostly influenced by time, as more recent reports consistently show higher acceptance rates indicating that HCWs became more accepting of the vaccine as it became more available and its side effects profile more elucidated, both of which are time-oriented concerns.

The variability between nurses and physicians in terms of vaccine acceptance rates were reported in a multitude of reports for both COVID-19 and seasonal flu vaccines [9,26,45]. A study conducted in Cyprus demonstrated that the greater majority of nurses are vaccine-hesitant and called for the need for public health policies targeting such vital HCW groups [46]. These observations are concerning since nursing staff have higher frequencies and longer durations of contact with patients, especially during the COVID-19 pandemic [26], thus being able to shape/influence their patients’ attitudes towards the vaccine. These tendencies might be attributed to the predominance of females within nursing, as women were less prone to take the COVID-19 vaccine; in fact, being a female predicts lower willingness to take the vaccine or support mandatory vaccination policies [27,35,47]. 

Such observations are striking as studies using the health belief model demonstrated that females are more likely to adopt, support and comply with preventive measures in response to health hazards such as COVID-19 [48]. It is plausible that such results may be attributed to the fact that more male HCWs are affected by COVID-19, which renders them more appreciative/receptive of the preventive value of vaccination [25,27] or the increased vaccine skepticism demonstrated by women [49]. The latter was demonstrated among our cohort as females were less likely to have COVID-19 transmission concerns and were less inclined to vaccinate previously infected individuals. Moreover, females, most notably married ones, may consider the effect of the vaccine on their fertility, breastfeeding, and children [15,30]. These gender differences suggest that efforts made to influence the decision to adopt the COVID-19 vaccine should be tailored differently on the basis of gender.

We observed that HCWs previously vaccinated for seasonal flu were more likely to accept the COVID-19 vaccine and are more willing to register in special COVID-19 vaccination platforms in cases where they did not yet take any doses. Such observations are consistent with the literature as being vaccinated against seasonal flu was significantly associated with COVID-19 vaccine acceptance and is also a predictor of acceptance [26]. This same finding was reported among the recently surveyed Jordanian public [50]. On a different note, physicians were significantly more likely to understand the efficacy, side effect profile, and dose effectiveness of the COVID-19 vaccine in comparison to nurses, thus presenting more positive attitudes towards its uptake and promoting it to those eligible; a trend that was documented throughout the literature irrespective of HCW work designation [30]. However, the suboptimal knowledge of nurses who are highly educated and clinically trained HCWs is not a result of their formal education but rather the inability to search and critique information [15]. Another reason might be due to the limited participation of Jordanian nurses in research-related activities, and so their ability to explore and critique the literature on recent medical advancements might be hindered. Despite their scientific and medical training, HCWs are a heterogeneous group and are not experts in the field of vaccination [24]; therefore, gaps within their immunological sciences may have also contributed to the aforementioned differences.

Our results showed that HCWs vaccine hesitancy resulted from a lack of confidence, lack of knowledge, and lack of trust in the efficacy of the COVID-19 vaccine. Barriers to HCWs acceptance of vaccines are consistent yet heterogeneous in frequency across the literature [23,25,28,31,33,34,51]. The most common reasons contributing to vaccine hesitancy include concerns regarding safety and efficacy of the vaccine, distrust in the health system and pharmaceutical companies, concerns regarding the rapid development of the vaccine, and pressure from personal communications [28,33,34]. Vaccine hesitancy appears to be multifactorial and consistent across all vaccines, yet greater barriers are expected for a vaccine developed with newer technologies [34]. The observed state of skepticism and distrust are the major barriers to COVID-19 vaccine uptake, which seem to be caused by the lack of information. Therefore, further studies are required to investigate the reasons for this distrust while governmental efforts are warranted to supply comprehensive knowledge to HCWs and the community alike with regards to the vaccine, its development, and clinical endpoints. However, due to the vaccine’s novelty, HCWs are expected to accept the vaccine at a later time [23].

It appears that whether attitudes towards the vaccine are strictly rational is a matter of controversy. Sun et al. (2021) demonstrated that the general health, personality, and physical and mental health statuses of HCWs did not affect their intention to vaccinate [39]. On the other hand, Szmyd et al. (2021) demonstrated that depression is a negative predictor of vaccine uptake while stress is a positive inducer of vaccine uptake [38]. Aversion to the vaccine can be induced by the spread of false information through anti-vaccination social media and the internet. A variety of conspiracy theories, more compelling than published scientific facts, have been shown to have impacted non-HCW groups due to their lack of knowledge or expertise within virology [38].

Our study demonstrated that HCWs derive their vaccine information primarily from governmental sources, followed by the internet and social media. This was in tandem with the literature as multiple reports show that governmental sources and online news agencies are the most trusted and utilized sources on COVID-19 vaccine information and updates [15,25,28,34]. Nonetheless, across low to middle-income countries, it is reported that the greatest amount of trust of the general public was allocated to healthcare workers and close social circles, while governmental sources and celebrities were the least trusted [52].

The study falls prey to a multitude of limitations. The study’s cross-sectional design may only provide a snapshot of the true acceptance rates among Jordanian HCWs and is not suitable for causal relationships. The use of a questionnaire with closed-ended answers might have missed some pertinent concerns. The small sample size and the variable response rate for each question might hinder the generalizability of the results. Longitudinal studies are warranted to investigate the temporal changes of vaccine acceptance rates. Social desirability, selection and recall biases are not uncommon within such kinds of observational designs. Moreover, the study setting might have limited a wider range of responses that may have been exhibited by public and governmental hospitals.

A myriad of solutions can be suggested to policymakers in order to tackle vaccine hesitancy among both HCW and the general public. The case for vaccine mandates (i.e., vaccine passports) has proven to be effective in Lithuania [53]. Such mandates work well for small to medium scale nations. Moreover, the government should expend more efforts into investigating the reasons behind vaccine hesitancy among HCWs and providing short term incentives to encourage vaccination. Moreover, policymakers should distribute vaccine-related information through trustworthy mediums (i.e., organizations) and not politicians [54]. Additionally, concerned authorities should control false information heavily advertised through social media and the internet. Long-term wise, HCWs should receive extensive training in virology and vaccinology to ensure their understanding of landmark developments in such fields.

## 5. Conclusions

The study portrays that Jordanian HCWs hesitancy towards the COVID-19 vaccine is low. However, the concerns of specific subgroups such as females and nurses should be addressed in order to maximize vaccine coverage. Ergo, it is urgent for independent committees and trusted authorities to provide interventions (e.g., workshops, online seminars, and tailored messages) and information regarding the vaccine’s safety and efficacy. Moreover, seamless communication should be facilitated between HCWs and healthcare authorities using all feasible channels.

## Figures and Tables

**Table 1:** Demographic characteristics of healthcare workers stratified by work designation.

		Physicians (*n* = 215)		Nurses (*n* = 149)		Total		*p*-Value
Variable (Total *)		Frequency	%	Frequency	%	Frequency	%	
Age groups in years (*n* = 351)	Less than 30	120	56.9%	80	57.1%	200	57.0%	1.000
	30 and above	91	43.1%	60	42.9%	82	23.4%	
	Total	211	100%	140	100%	351		
Gender (*n* = 364)	Male	110	51.2%	25	16.8%	135	37.1%	<0.001
	Female	105	48.8%	124	83.2%	229	62.9%	
	Total	215	100%	149	100%	364	100%	
Marital status (*n* =361)	Married	118	54.9%	79	53%	197	54.6%	0.620
	Not married	94	44.3%	70	47%	164	45.4%	
	Total	212	100%	149	100%	361	100%	
Experience (*n* = 362)	10 years or less	160	74.4%	96	65.3%	256	70.7%	0.061
	More than 10 years	55	25.6%	51	34.7%	106	29.3%	
	Total	215	100%	147	100%	362	100%	
Seasonal flu vaccination (*n* = 238)	Yes (regular and not regular)	118	84.3%	79	80.6%	197	82.8%	0.460
	No	22	15.7%	19	19.4%	41	17.2%	
	Total	140	100%	98	100%	238	100%	
Previous adverse effects to seasonal flu (*n* = 195) **	Yes	22	19%	23	29.1%	45	23.1%	0.099
	No	94	81%	56	70.9%	150	76.9%	
	Total	116	195	79	100%	195		
Registered in the special platform for COVID-19 vaccination (*n* = 345)	Yes	141	70.5%	59	29.5%	190	55.1%	<0.001
	No	49	33.8%	96	66.2%	155	44.9%	
	Total	190	100%	155	100%	345		
Source of information about COVID-19 (***)	Ministry of Health	120	55.8%	76	51%	186	51.1%	0.013
	Internet including Blogs	79	36.7%	37	24.8%	116	31.9%	0.016
	Friends	37	17.2%	19	12.8%	56	15.4%	0.216
	Radio/TV	33	15.3%	15	10.1%	48	13.2%	0.143
	Social media	62	28.8%	43	28.9%	105	28.8%	0.996
	Newspaper/magazine	19	8.8%	8	5.4%	27	7.4%	0.214
	Self-reading and searching	54	25.1%	14	9.4%	68	18.7%	<0.001

* The total might differ for each question depending on the number of individuals who answered this particular question. ** Those who can answer this question are 238 because those who did not receive the seasonal flu vaccine will not have the option to answer this question. *** the participants had the chance to choose more than one option.

**Table 2:** Participants’ responses stratified by occupation.

	Physicians (*n* = 215)				Nurses (*n* = 149)				*p*-Value	Adj. *p*-Value for Gender
	Agree		Disagree		Agree		Disagree			
**Practice**	*N*	%	*N*	%	*N*	%	*N*	%		
Have you been regularly vaccinated against the seasonal flu	118	84.3	22	15.7	79	80.6	19	19.4	0.489	0.932
Have you registered in the special platform for COVID-19 vaccination?	141	65.6	59	27.4	49	32.9	96	64.4	<0.001	<0.001
**Attitudes**										
I would register on the special platform if I have already not done so.	30	53.6	26	46.4	16	16.8	79	83.2	<0.001	<0.001
Seasonal flu vaccines should be given to all health care workers.	129	92.1	11	7.9	75	77.3	22	14.8	0.002	0.014
How concerned are you that you or a family member could become infected with COVID-19 this year?	162	78.3	45	21.7	101	69.7	44	30.3	0.081	0.203
COVID-19 vaccine should be given to all health care workers.	181	88.7	23	11.3	89	61.4	56	38.6	<0.001	<0.001
There is no need to vaccinate people who have been infected with COVID-19.	74	36.1	131	63.9	77	52.4	70	47.6	0.003	0.001
I will take the COVID-19 vaccine when it becomes available.	170	83.3	34	16.7	63	42.6	85	57.4	<0.001	<0.001
I will recommend the COVID-19 vaccine to groups of people who are considered eligible as per the national health vaccine safety protocol.	187	92.6	15	7.4	81	55.9	64	44.1	<0.001	<0.001
**Knowledge**										
The COVID-19 vaccine is very effective in preventing COVID-19 infection and its related serious morbidity and mortality.	181	87.9	25	12.1	81	54.4	66	44.3	<0.001	<0.001
Two doses of the COVID-19 vaccine should be given to prevent infection.	186	90.7	19	8.8	93	62.8	55	37.2	<0.001	<0.001
Receiving the COVID-19 vaccine would lead to long lasting immunity.	95	46.3	110	53.7	63	42.6	85	57.4	0.516	0.788
The COVID-19 vaccine would not cause serious adverse side effects.	132	65.0	71	35.0	58	38.9	89	60.5	<0.001	<0.001
The COVID-19 vaccine would eliminate the need for regular protective measures such as, facemasks, social distancing, and frequent hand washing.	44	21.3	163	78.7	43	28.9	104	70.7	0.103	0.128
Do you have sufficient information about COVID-19 vaccine?	85	66.9	42	33.1	41	42.3	56	57.7	<0.001	0.022
If no, would you like to attend a session about COVID 19?	32	74.4	11	25.6	36	64.3	20	35.7	0.382	0.257

**Table 3:** Participants’ responses stratified by gender.

	Male (*n* = 135)				Female (*n* = 229)				*p*-Value	Adj. *p*-Value for Occupation
	Agree		Disagree		Agree		Disagree			
**Practice**	*N*	%	*N*	%	*N*	%	*N*	%		
Have you been regularly vaccinated against the seasonal flu?	77	87.5	11	12.5	120	80.0	30	20.0	0.158	0.194
Have you registered on the special platform for COVID-19 vaccination?	85	65.4	45	34.6	105	48.8	110	51.2	0.004	0.514
**Attitudes**										
I would register on the special platform if I have already not done so.	14	33.3	28	66.7	32	29.4	77	70.6	0.694	0.257
Seasonal flu vaccines should be given to all health care workers.	81	92.0	7	8.0	123	82.6	26	17.4	0.052	0.454
How concerned are you that you or a family member could become infected with COVID-19 this year?	105	80.2	26	19.8	158	71.5	63	28.5	0.077	0.203
The COVID-19 vaccine should be given to all health care workers.	109	83.2	22	16.8	161	73.9	57	26.1	0.048	0.905
There is no need to vaccinate people who have been infected with COVID-19.	48	36.9	82	63.1	103	46.4	119	53.6	0.094	0.467
I will take the COVID-19 vaccine when it becomes available.	96	74.4	33	25.6	137	61.4	86	38.6	0.014	0.708
I will recommend the COVID-19 vaccine to groups of people who are considered eligible as per the national health vaccine safety protocol.	106	84.1	20	15.9	162	73.3	59	26.7	0.024	0.444
**Knowledge**										
The COVID-19 vaccine is very effective in preventing COVID-19 infection and its related serious morbidity and mortality	102	77.9	29	22.1	160	72.1	62	27.9	0.258	0.134
Two doses of the COVID-19 vaccine should be given to prevent infection	114	87.0	17	13.0	165	74.3	57	25.7	0.004	0.485
Receiving the COVID-19 vaccine would lead to long lasting immunity.	64	49.2	66	50.8	94	42.2	129	57.8	0.223	0.266
The COVID-19 vaccine would not cause serious adverse side effects.	79	61.7	49	38.3	111	50.0	111	50.0	0.035	0.595
The COVID-19 vaccine would eliminate the need for regular protective measures such as, facemasks, social distancing, and frequent hand washing.	29	22.1	102	77.9	58	26.0	165	74.0	0.445	0.815
Do you have sufficient information about COVID-19 vaccine?	60	71.4	24	28.6	66	47.1	74	52.9	<0.001	0.034
If no, would you like to attend a session about COVID 19?	17	70.8	7	29.2	51	68.0	24	32.0	1.000	0.615

## Data Availability

Data will be provided from the corresponding author at a reasonable request.
